# PLGA-based gene delivering nanoparticle enhance suppression effect of miRNA in HePG2 cells

**DOI:** 10.1186/1556-276X-6-447

**Published:** 2011-07-12

**Authors:** Gao Feng Liang, Yan Liang Zhu, Bo Sun, Fei Hu Hu, Tian Tian, Shu Chun Li, Zhong Dang Xiao

**Affiliations:** 1 State Key Laboratory of Bioelectronics, School of Biological Science and Medical Engineering, Southeast University, Nanjing, 210096, China

## Abstract

The biggest challenge in the field of gene therapy is how to effectively deliver target genes to special cells. This study aimed to develop a new type of poly(D,L-lactide-co-glycolide) (PLGA)-based nanoparticles for gene delivery, which are capable of overcoming the disadvantages of polyethylenimine (PEI)- or cationic liposome-based gene carrier, such as the cytotoxicity induced by excess positive charge, as well as the aggregation on the cell surface. The PLGA-based nanoparticles presented in this study were synthesized by emulsion evaporation method and characterized by transmission electron microscopy, dynamic light scattering, and energy dispersive spectroscopy. The size of PLGA/PEI nanoparticles in phosphate-buffered saline (PBS) was about 60 nm at the optimal charge ratio. Without observable aggregation, the nanoparticles showed a better monodispersity. The PLGA-based nanoparticles were used as vector carrier for miRNA transfection in HepG2 cells. It exhibited a higher transfection efficiency and lower cytotoxicity in HepG2 cells compared to the PEI/DNA complex. The N/P ratio (ratio of the polymer nitrogen to the DNA phosphate) 6 of the PLGA/PEI/DNA nanocomplex displays the best property among various N/P proportions, yielding similar transfection efficiency when compared to Lipofectamine/DNA lipoplexes. Moreover, nanocomplex shows better serum compatibility than commercial liposome. PLGA nanocomplexes obviously accumulate in tumor cells after transfection, which indicate that the complexes contribute to cellular uptake of pDNA and pronouncedly enhance the treatment effect of miR-26a by inducing cell cycle arrest. Therefore, these results demonstrate that PLGA/PEI nanoparticles are promising non-viral vectors for gene delivery.

## Introduction

MicroRNAs (miRNAs) are small, highly conserved, non-coding RNAs that regulate gene expression at the post-transcriptional level. They involve in various cellular mechanisms including development, differentiation, proliferation, and apoptosis. The pivotal roles of these miRNAs in human cancers have been discovered [1,2], and the therapeutic applications of miRNA have been developed using various viral vectors [3,4].

However, the disadvantages of viral vectors limited their application in gene delivery, such as immunogenic/inflammatory responses, low loading capacity, large scale manufacturing, and quality control [5]. Consequently, more attention have been paid on non-viral gene delivery vectors in recent years, such as liposomes (lipoplexes), polycationic polymers (polyplexes), and organic or inorganic nanoparticles (nanoplexes) [6]. To enhance gene delivery effect, various cationic complexes have been developed for delivering plasmid DNA, antisense, or siRNA into cells [7-9]. Poly(D,L-lactide-co-glycolide) (PLGA) were extensively assessed for their ability of delivering variety of therapeutic agents [10-12]. PLGA nanoparticles were shown to escape from the endo-lysosomal compartment to the cytoplasmic compartment and release their contents over extended periods of time [13]. These features rendered PLGA nanoparticles as potential tool for gene delivery efficiently.

Polyethylenimine (PEI) is water-soluble, linear, or branched polymers with a protonable amino group [14,15]. Due to their high cationic charge density at physiological pH, PEIs are able to form non-covalent complexes with DNA, siRNA, and antisense oligodeoxynucleotide. Therefore, PEIs hold a prominent position among the polycationic polymers used for gene delivery [16-18]. The intracellular release of PEI/nucleic acids complexes from endosomes is considered as relying on the protonation of amines in the PEI molecule, which leading to osmotic swelling and subsequent burst of the endosomes. Moreover, PEIs also facilitate nucleic acid entry into the nucleus [19,20]. However, it has been reported that long PEI chains are highly effective in gene transfection, but more cytotoxic [14,21,22].

In order to overcome these hurdles in gene therapy and improve gene delivery efficiency, we developed novel non-liposome-based cationic polymers which are composed of PLGA as the core and cationic PEI as the shell. The biodegradable PLGA nanoparticles, modified with a polyplexed PEI coating, were tested by loading the expression vector (pDNA) of miR-26a, which is capable of inducing cell cycle arrest in HepG2 cells. In this study, nanoparticles of controlled size and persistent shape have been obtained by an emulsion evaporation method and characterized by transmission electron microscopy (TEM), dynamic light scattering (DLS), and energy dispersive spectroscopy (EDS). The nanoparticles have been determined by their physicochemical and biological properties. The formulated nanoparticles enhance cellular uptake of miRNA, pronounce upregulation of miR-26a, induce cell cycle arrest, and improve gene expression activity compared with PEI and commercial liposome. Furthermore, these particles can be easily fabricated and have a high transfection efficiency and low cell toxicity. Our results suggest a new approach for miRNA delivering by PLGA/PEI nanoparticles in gene therapy.

## Materials and methods

### Materials

Branched PEI (*M*_W_, 25 kDa) and poly(vinylalcohol) (PVA) were obtained from Sigma Aldrich (St. Louis, MO, USA). D,L-Lactide/glycolide copolymer (PLGA, lactic/glycolic molar ratio: 53/47; *M*_W_, 25 kDa) was purchased from Daigang Chemical Reagent Co., Ltd. (Jinan City, Shandong Province, China). Dulbecco's modified Eagle's medium (DMEM), fetal bovine serum (FBS), penicillin-streptomycin, trypsin, and Dulbecco's PBS were purchased from Invitrogen (Carlsbad, CA, USA), and pGFP-miRNA plasmid was constructed according to the methods described previously [23]. Other reagents were of analytical grade obtained from suppliers and used without purification.

### PLGA/PEI nanosphere synthesis

PLGA nanospheres were obtained by using water-in-oil-in-water solvent evaporation technique as described previously [24]. Briefly, 150 mg of PLGA polymer was dissolved in 1.5 ml of dichloromethane to yield a 10% (*w*/*v*) polymer solution. After 3 ml of a 7% (*w*/*v*) aqueous solution of PVA was added to the organic phase and emulsified at 10,000 × *g *using a homogenizer for 5 min. The resulting double emulsion was then poured into 50 ml of a 1% PVA solution and emulsified for 15 min. This resulted in the formation of a water/oil/water emulsion that was stirred for at least 12 h at room temperature, allowing the methylene chloride to evaporate. The resulting microspheres were washed twice in deionized water by centrifugation at 16,000 × *g *and freeze-dried.

Then, PEI aqueous solution was added in the aforementioned PLGA nanoparticle suspension, and incubated fifteen minutes.

### Nanoparticle characterization

PLGA nanoparticles, PLGA/PEI nanoparticles, and PLGA/PEI /pDNA complexes mean hydrodynamic diameters measurements were conducted by using Nano Particle Analyzer (Beckman Coulter, Fullerton, CA, USA). The mean hydrodynamic diameter was determined via cumulative analysis.

The zeta-potential (surface charge) of the polymers and polyplexes was determined at 25°C with a scattering angle of 90° using potential measurement analyzer (90 PLus, Brookhaven, Holtsville, NY, USA). Samples were prepared in PBS and diluted with deionized water to ensure that the measurements be performed under conditions of low ionic strength where the surface charge of the particles can be measured accurately.

The particle size and morphology of the PLGA nanoparticles, PEI-modified PLGA nanoparticles, and PEI-modified PLGA nanoparticles/miRNA complexes were characterized via transmission electron microscope (TEM, JEM-2100, JEOL, Tokyo, Japan).

Energy dispersive spectroscopic analysis (Oxford EDS, Oxford Instruments, Oxon, UK) was employed to perform the quantitative elemental analysis of the nanocomplex.

### Measurement of the interactions between miRNA and nanoparticles

Complexes were formed by diluting miRNA expression vector (pDNA) and the different amount of nanoparticles separately with 0.9% NaCl (pH 7.4). The nanoparticles at different concentrations were added to 1 μg green fluorescent protein(pPG-eGFP-miR) (GFP)-encoded miRNA vector solution, vortexed immediately at room temperature, and allowed to stand for 30 min to form PLGA/PEI/pDNA complexes. Then, the complexes were submitted to electrophoresis in 1%/TAE agarose gel at 90 V for 60 min. Images were acquired using a PeiQing gel imaging system (PeiQing, Shanghai City, China). GelRed (Biotium, Hayward, CA, USA), an ultra sensitive nucleic acid dye, was used to examine the interactions of DNA with the nanocomplex to determine optimal N/P ratio of the nanocomplex [25].

### Cytotoxicity studies

HepG2 (human hepatocellular carcinoma cell) cells were cultured in DMEM supplemented with 10% FBS, streptomycin at 100 μg/mL, and penicillin at 100 U/mL. Cells were maintained at 37°C in humidified and 5% CO_2 _incubator. The cytotoxicity of PLGA/PEI nanoparticles, complexed with or without pDNA, was determined in a separate set of experiments using MTT assay to detect changes in cell viability after an incubation time of 24 h. Cells were seeded in 24-well plates at an initial density of 2 × 10^4 ^cells/well for HepG2 in 0.5 mL of growth medium and incubated for 24 h prior to the addition of PLGA/PEI and PLGA/PEI/DNA at different N/P ratio. Untreated cells were taken as control with 100% viability. Triton X-100 1% (SPI Supplies, West Chester, PA, USA) was used as positive control of cytotoxicity.

### *In vitro *gene transfection and quantification study

Before GFP transfection assay, cells were seeded in 24-well plates at a density dependent of the cell line in DMEM with 10% FBS. When the cells were at 50% to 70% confluence, the medium in each well was replaced with fresh normal medium or medium containing naked pGFP, lipo2000, or PLGA/PEI/DNA complex under standard incubator conditions. After 48 h, cells harboring an expressing integrant were viewed by fluorescence microscopy based on GFP.

The analysis of transfection efficiency was performed using a flow cytometer (BD Biosciences, Mountain View, CA, USA). Cells were first washed with PBS and detached with 0.2 mL of 0.25% trypsin. Growth medium was then added, and the cells suspension was centrifuged at 1,000 rpm for 5 min. Two further cell-washing cycles of resuspension and centrifugation was carried out in PBS before fixation in 0.4 mL of 75% ethanol. The percentage of cells expressing GFP was then determined from 10,000 events and reported as a mean ± standard deviation (SD) of three samples.

### miRNA expression and cell cycle study

Gene-specific primers and reverse transcriptase were used to convert mature miRNA to cDNA [26], DNase-treated total RNA (20 μl of total volume) was incubated with 1 μl of 10 mM reverse transcription primers listed in Table 1. The reaction was heated to 80°C for 5 min to denature the RNA and then cooled to room temperature quickly, after that the remaining reagents (5 × buffer, primescript RTase (TaKaRa, Dalian, China), dNTPs, DTT, and RNase inhibitor(SunshineBio, Nanjing, China) were added as specified according to the manufacturer's protocol. The reaction proceeded for 45 min at 42°C followed by 5 min incubation at 85°C to inactivate the reverse transcriptase. cDNA may be stored at -20°C or -80°C.

**Table 1:** Gene-specific primers used to amplify the miRNAs

Gene	Forward primer (5' → 3')	Reverse primer (5' → 3')
RT-miR-26a	CTCAACTGGTGTCGTGGAGTCGGCAATTCAGTTGAGAGCCTATC	
miR-26a	ACACTCCAGCTGGGTTCAAGTAATCCAGGA	TGGTGTCGTGGAGTC
U6	GCTTCGGCAGCACATATACTAAAAT	CGCTTCACGAATTTGCGTGTCAT

Then, real-time quantitative polymerase chain reaction (PCR) was performed to evaluate miR-26a expression in HepG2 cells after 3 days transfection using standard protocols on an Applied Biosystems 7500 Sequence Detection System (Applied Biosystems, Foster City, CA, USA). Briefly, 1.25 μl of cDNA was added to 10 μl of the 2 × SYBR green PCR master mix (TaKaRa, Dalian, China), 200 nM of each primer and water to 20 μl. The reactions were amplified for 15 s at 95°C and 1 min at 60°C for 40 cycles. The thermal denaturation protocol was run at the end of the PCR to determine the number of the products that were present in the reaction. Reactions are typically run in triple. The cycle number at which the reaction crossed an arbitrarily placed threshold (*C*_t_) was determined for each gene and the relative amount of each miRNA. Target gene expression was normalized to the expression of the housekeeping gene U6 for each sample. Data were analyzed using the  method [27].

Cell cycle profiles of nanoparticles transfected HepG2 cells were performed using a FACS Calibur and with CellQuest™ software (BD Biosciences, Mountain View, CA, USA). HepG2 cells (5 × 10^4 ^per well) were seeded into 24-well plates. Cells were treated with different formulations at a concentration of 1 μg miRNA in serum containing medium at 37°C for 72 h. Cells were washed once with PBS and then fixed in 75% ethanol and, after fixation, stained with PI according to manufacturer's instructions.

### Statistical analysis

All statistical analyses were performed by Student's *t *test. Data were expressed as means ± SD. Results were considered statistically significant when the *p *value was less than 0.05.

## Results and discussions

### Particle size and surface morphology of the nanoparticles

Particle size and zeta potential have been demonstrated to play important roles in determining the level of cellular and tissue uptake; researchers have also conducted to investigate the size effect on the gene delivery efficiency. PLGA nanoparticles with smaller size below 100 nm have been proved to gain higher gene transfection efficiency than those of 200 nm [28,29].

**Figure 1:**
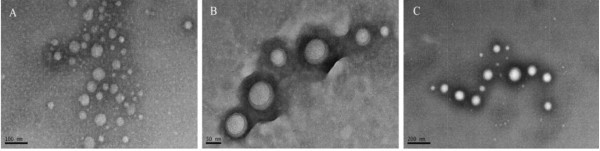
**TEM images of free PLGA nanoparticle; PLGA/PEI, and PLGA/PEI/pDNA**. Scale bars: 100, 50, and 200 nm, respectively.

In this study, PLGA-based nanoparticle was developed and characterized using TEM, DLS, and EDS. Figure 1 showed different characteristics of PLGA nanoparticles with PEI or PEI/pDNA. The diameter of the nanoparticles/DNA complexes ranged from 45 to 70 nm at the determined nanoparticles/DNA N/P ratios. Slight size changes were observed following coating of the biodegradable PLGA nanoparticles with PEI or PEI/pDNA. The mean diameter of the sole PLGA nanoparticles was approximately 50 nm, whereas the sizes of complexed PLGA nanoparticles containing PEI or PEI/pDNA are increased to about 57 or 60 nm, respectively. These results are consistent with our DLS observations and indicate that the PLGA nanoparticle size may be increased with PEI or polyplexed PEI/pDNA coating.

Zeta potential is an indicator of the surface charge of nanoparticles/DNA complexes. The positive charges on the surface of the complexes can help the nanoparticles bind tightly to the negatively charged cellular membrane, therefore facilitating their entry into the cells by endocytosis. In this study, zeta potential values of the PLGA nanoparticles and PLGA/PEI nanoparticle and PLGA/PEI/pDNA complexes (at various N/P ratios ranging from 1 to 10) were measured. The values of the PLGA nanoparticles, PLGA/PEI nanoparticles, and PLGA/PEI/pDNA complexes are -21.4, 29.4, and 23.7 mV, respectively. As shown in Table 2, the pure PLGA nanoparticles represent negative potential due to the existence of carboxyl. PEI is postulated on the surface of PLGA nanoparticles due to the electrostatic interaction of cationic PEI molecules with anionic PLGA polymers. Introduction of PEI increase significantly the zeta potential of the nanoparticles. Again, pDNA are adsorbed on the surface of PLGA/PEI nanoparticles due to the electrostatic interaction of negatively charged pDNA with positively charged PLGA/PEI nanoparticles. The zeta potential of the complexes increase in parallel with the nanoparticles/pDNA N/P ratio, ranging from -21.4 to +23.7 mV, which results in a good affinity to cell surface.

**Table 2:** Size distribution, zeta potential, and elementary analysis of the nanoparticles

Type of formulation	Size (nm)	Zeta potential (mV)	Atom% - O	Atom% - N	Atom% - P
PLGA	50 ± 6	-21.4 ± 1.8	49% (± 0.74)	0% (± 0)	0% (± 0)
PLGA-PEI	57 ± 7	29.4 ± 2.6	42% (± 0.63)	4.7% (± 0.32)	0% (± 0)
PLGA-PEI/pDNA(N/P = 6)	60 ± 7	23.7 ± 2.3	41% (± 0.98)	4.1% (± 0.43)	1.1% (± 0.13)

EDS is capable of providing both qualitative and quantitative information about the presence of different elements in nanoparticles. In this study, the surface modification of PLGA nanospheres with PEI or PEI/pDNA was confirmed by EDS. Because PEI contains the nitrogen element and pDNA contains phosphorus element, but PLGA contains neither of them, the nitrogen that is detected in PLGA/PEI nanoparticles is an evidence for the existence of PEI on the PLGA nanoparticles surface (Table 2). By detecting of phosphorus PLGA/PEI/pDNA nanocomplex, we have shown that pDNA has been successfully adsorbed on the PLGA/PEI surface. From the combination of the aforementioned data, it can be inferred that the PEI or pDNA is completely complexed with the nanoparticles by ionic binding.

### Characterization of nanoparticles/pDNA complexes

**Figure 2:**
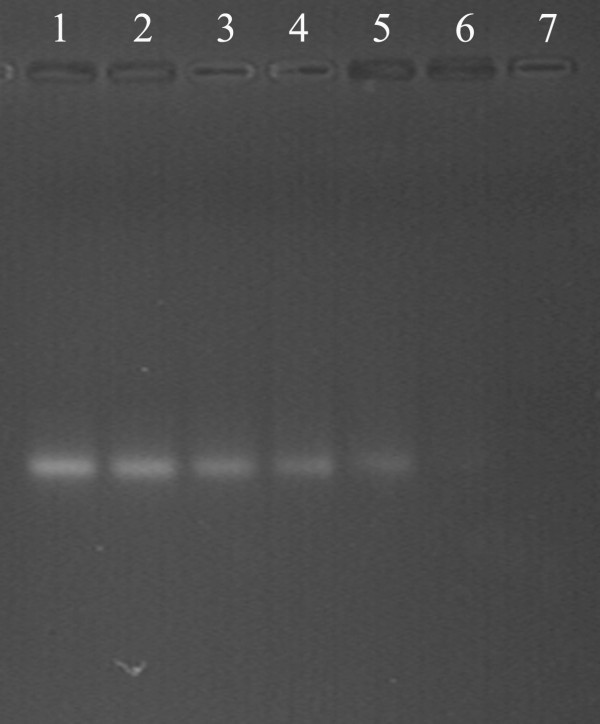
**Agarose gel electrophoresis assay of PLGA/PEI/pDNA nanocomplexes**. Lane 1, pDNA alone; lanes 2 to 7, PLGA/PEI /pDNA complexes N/P ratio 1, 2, 3, 4, 6, and 10.

To explore complex formation of pDNA and PLGA/PEI nanoparticles, PLGA/PEI nanoparticles were mixed with pDNA at various ratios. Complex formation was assessed by a gel retardation assay. The pDNA was vortexed with the nanoparticles in nuclease-free water at different N/P ratios. As shown in Figure 2, the nanoparticles are able to fully retard the mobility of DNA in agarose gel when the N/P ratio is 10:1 or higher than 10:1. While N/P is lower than 10:1, the retardation is not full. DNA bands are visible in N/P complexes in lanes 2 to 4 (Figure 2), indicating the presence of free DNA in nanocomplexes of 1 and 4 N/P ratios. This result is comparable with the band observed in lane 1, which contain only free plasmid. Few free DNA bands was observed in subsequent lanes of N/P ratios 6 and 10, indicating complete complexation of all free plasmid. The results indicate that the nanocomplexes can be easily prepared by simply mixing cationic polymer and DNA solution, and the results also showed that the optimal N/P is 6:1.

### *In vitro *gene expression assay

**Figure 3:**
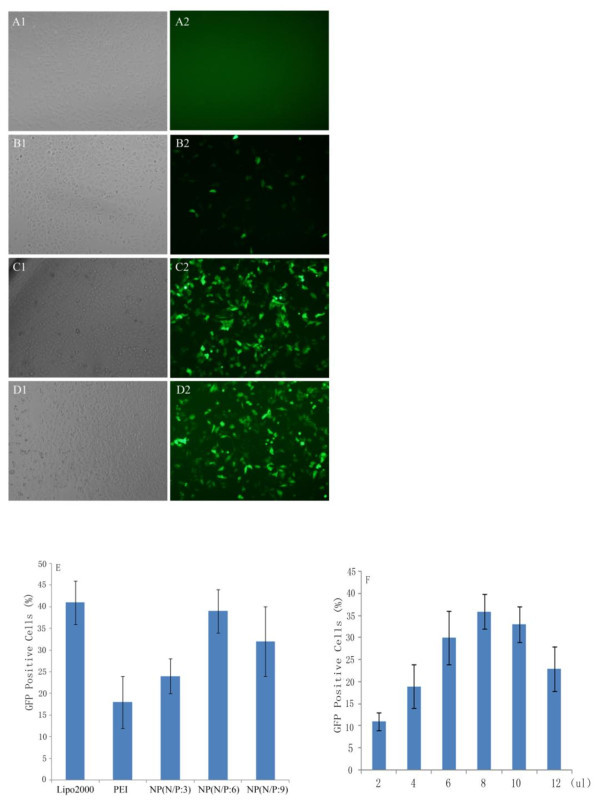
**GFP expression in HepG2 cells transfected with different transfection reagents**. (**A**-**D) **Fluorescent and bright-field images of green fluorescent protein expression in HepG2 cells for PLGA-based polyplexes (N/P ratio 6) with control 25 kDa PEI (N/P ratio 5) and lipo2000. (A) naked DNA, (B) PEI, (C) PLGA/PEI, (D) lipo2000. The images were obtained at magnification of 100×. (**E**) Transfection efficiency of the nanocomplex determined by flow cytometry analysis at different N/P ratio (*n *= 3). The transfection reagents/DNA complexes were prepared at their optimal condition. (**F**) Transfection efficiency of the nanocomplex determined by flow cytometry analysis at 2 μg pDNA mixed with different volume of nanocomplex (4 μg/μl). The nanoparticles/DNA complexes were prepared at optimal N/P ratio (*n *= 3).

It is clear that gene delivery is dependent on DNA/vector uptake efficiency. Fluorescent proteins, such as GFP, are usually used to label non-viral vectors for measuring the uptake efficiency. In this study, nanoparticle/pGFP-miR-26a complexes were employed for the assessment of the transfection efficiency in HepG2 cells. Firstly, HepG2 cells were transfected by 2 μg of pDNA complexed with 100 μl polymer at different nanoparticles/pDNA N/P ratios. The nanocomplexes were further evaluated for their transfection efficiency in cells by screening the GFP signals with flow cytometry. The results showed that transfection efficiency reached the optimum at nanoparticles/DNA N/P ratio 6 and further decreased till the N/P ratio reached 9 (Figure 3E). It is suggested that insufficient surface potential of complexes at low N/P ratios resulted in lower GFP expression, whereas at high N/P ratios, induction of complexes undoubtedly result in cytotoxicity because of the excessive PEI in the nanocomplex.

Next, transfection efficiency of the nanoparticles was compared to other different vehicle. As shown in Figure 3, GFP expression is hardly detected when transfection was mediated by naked pDNA, which was used as negative control. The results have demonstrated that the naked GFP is difficult to be directly internalized by the cells. In contrast, GFP-contained nanoparticles facilitate the cell endocytosis. Figure 3E also showed that transfection efficiency of nanoparticles is comparable to the commercial liposomes, and obviously better than the sole PEI particles.

Furthermore, PLGA/PEI nanoparticles display the transfection efficiency in a dose-dependent manner (Figure 3F). The uptake efficiency of nanoparticles by cells, assayed on 48 h after incubation, was higher at lower nanoparticle concentration. However, the transfection efficiency deceased when the nanoparticle concentration is further increasing due to the potential cytotoxicity.

Furthermore, we studied the stability of nanoparticles/pDNA in serum. Results suggested that transfection efficiency of the nanocomplexes is higher than lipo2000 in the presence of serum (data not shown). Meanwhile, the GFP expression at the 48 h of incubation was significantly higher than at 24 h. While the nanoparticle/DNA in the medium was washed away at 24 h after transfection, after another 24 h, GFP signals were not obviously enhanced (data not shown). The data indicate that the increasing GFP expression results from the sustained nanoparticle uptake effect of the transfected cells. It was further proved that the nanoparticles have good biocompatibility in serum.

### *In vitro *cytotoxicity

**Figure 4:**
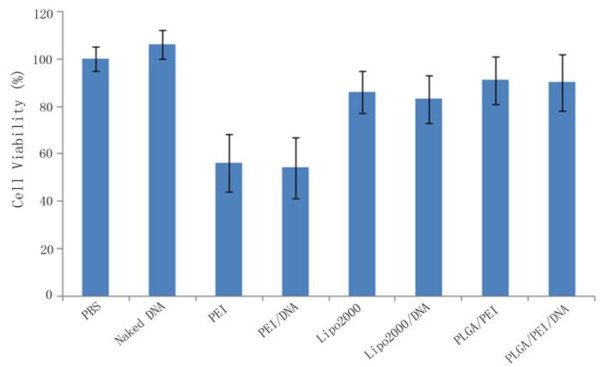
**Viability of HepG2 cells 48 h posttreatment with one of above transfection reagents**. Values are the mean average ± SD of 3 wells applied with the same reagents.

MTT assay has been widely used for cell proliferation and biochemical toxicity testing. In this study, MTT assay was used to investigate the cytotoxicity of nanoparticles/pDNA complexes on HepG2 cells. Figure 4 showed that the cytotoxicity of PLGA/PEI (N/P 6) nanoparticles is remarkably lower on HepG2 cells, compared with the pure PEI, and close to commercial liposome. Indeed, PLGA/PEI/pDNA showed above 90% cell viability at N/P 6. By contrast, cell viability dropped down about 54% and 86% in the presence of PEI and liposome, respectively; there is little effect on cell viability observed with or without DNA, and this confirmed that High Molecular Weight (HMW) PEI aggregates on the cell surface, inducing lysosomal breakdown and mitochondrial damage, thereby affecting cell viability [30]. Nanoparticles of PLGA as core, coated with PEI, presented in this study, effectively improve the stability of nanocomposites and avoid the release of toxic free PEI in the cells after delivery of the miRNA vector.

### miR-26 expression and cell cycle induction

**Figure 5:**
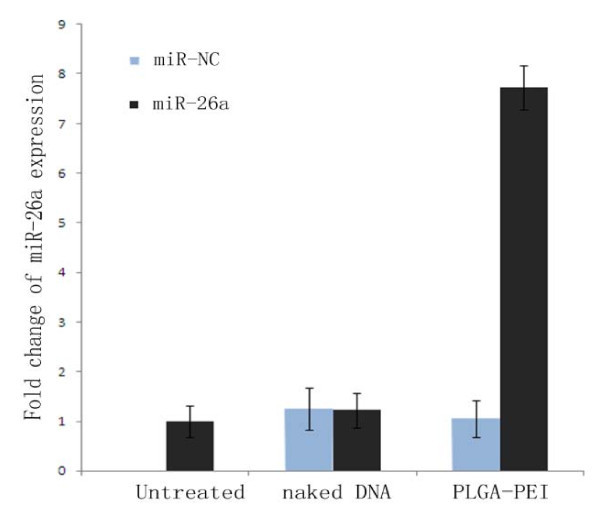
**Relative expression change of miR-26a level**. (1) Untransfected group; (2) naked DNA group; (3) PLGA/PEI transfected group. *P *< 0.05 compared with groups naked DNA and PLGA/PEI nanoparticles.

To examine the biological activities of miR-26a delivered by nanoparticles/pDNA in HepG2 cells, the expression levels of miR-26a were detected by qRT-PCR in transfected HepG2 cells. Forty-eight hours after transfection, HepG2 cells were harvested and total RNA was extracted for monitoring miRNA expression. Real-time quantitative RT-PCR results show that the miR-26a level in the transfected cells is increased by 7.73-folds compared with untransfected cells (*p *< 0.05) (Figure 5). In contrast, the expression level of miR-26a shows no obvious change in cells transfected with negative control (miR-NC) and naked pDNA (*p *> 0.05).

**Figure 6:**
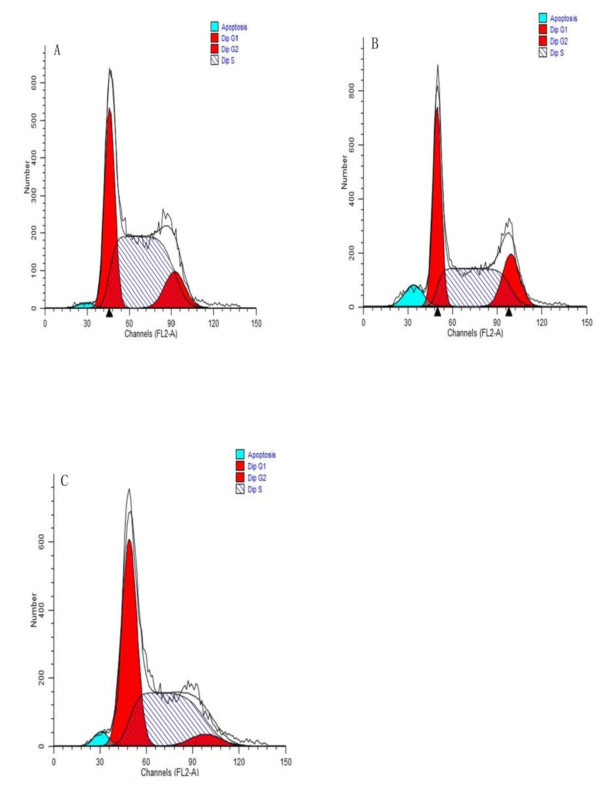
**Cell cycle profiles of PLGA-based nanoparticles transfected HepG2 cells**. Numbers in Table 3 indicate the percentage of cells remaining in each phase of cell cycle. (**A**) Untreated group; (**B**) miR-NC contained nanoparticles transfected HepG2 cells; (**C**) miR-26a contained nanoparticles transfected HepG2 cells. Table 3 numbered cell cycle profiles corresponding to this figure.

The miR-26a-containing PLGA-based nanocomposites significantly increase the expression level of miR-26a and inhibit the cell cycle progression by induction of G1 phase arrested in transfected HepG2 cells, whereas the effect in control groups (miRNA negative control transfected cells) were not detectable. Figure 6C indicates that cell populations with enforced miR-26a expression were characterized by significantly increased numbers of cells arrested in G1, which is more than that of tumor cells treated with miR-NC containing PLGA nanocomplex or untreated control (Table 3). Kota et al. have demonstrated that upregulation of miR-26a expression results in the inhibition of cancer cell proliferation, induction of tumor-specific apoptosis, and enhancement of antitumor activity [3]. In this study, we have developed a PLGA-based nanocomplex and tested its gene delivery ability on HepG2 cells. Our studies demonstrate that PLGA nanocomplex facilitated cellular uptake and enhanced gene expression activity.

**Table 3:** Numbered cell cycle corresponding to Figure 6

Phase	Untreated	miR-NC	miR-26a
G1	31.32	35.72	46.76
G2	11.32	16.68	5.36
S	57.36	45.60	47.88

Researchers have reported that HMW cationic PEI as a transfection reagents showed higher cytotoxicity [21,22,31,32]. In our studies, the formulation of PLGA-based nanoparticle significantly reduces the cytotoxicity of the PEI. We have demonstrated that PLGA/PEI nanocomplex shows higher gene transfection efficiency and better serum compatible than Lipofectamine2000 or PEI. As for the possible reasons, we speculated that the enhanced transfection effect may be related to the interaction between PLGA and PEI. In the given nanoparticles mentioned above, PLGA showed a better biocompatibility than the lipids. Obviously, the amino groups of PEI play an important role in determining the biological characteristics of the nanocomplexes. To our knowledge, there are few scientific articles describing solid preformed PEI-based nanoparticles and their cellular applications. Moreover, in the most of these studies, PEI is complexed through cooperative electrostatic interactions with other anionic polymers or converted into nanoparticles by introducing ionic and covalent crosslinkers without any addition of other polymers.

Furthermore, the properties of the nanoparticles we prepared can be easily controlled. Their size can be tuned by modulating experimental conditions of preparation, and the surface charge can be adjusted by changing the PEI amount introduced into the complex. Finally, cytotoxicity studies showed that the PLGA/PEI/DNA complex exhibited less cytotoxicity than HMW PEI and liposome. In the tested cell line, the transfection efficiencies mediated by PLGA/PEI increased with the increase of nanoparticles/DNA NP ratio in the presence or absence of serum.

## Conclusion

In this study, we developed a PLGA-based nanoparticles polyplexed with miRNA expression vector as a potential approach to deliver genes with non-viral vectors. The biodegradable nanoparticles can efficiently deliver nucleic acid to human hepatocellular carcinoma cells and enhance the effect of functional miRNA delivered, such as the cell cycle suppressor miRNA26a. Importantly, it is proved that serum cannot inhibit the transfection activity of this nanoparticle. These PLGA-based nanoparticles also display an improved safety profile in comparison with high molecular weight PEIs and liposome because of the lower cytotoxicity of the polyplex formulations. This study presents an effective gene delivery vehicle, PLGA-based nanoparticle, which may contribute to the gene therapy for tumor and other miRNA-related diseases such as diabetic, cardiovascular disease, and neurodegenerative diseases.

## Authors' contributions

GFL designed the experiment, carried out the molecular biologic studies and drafted the manuscript. YLZ carried out the preparation and characterization of nanoparticles drafted the manuscript. ZDX conceived of the study, and participated in its design and coordination. All authors read and approved the final manuscript

## Competing interests

The authors declare that they have no competing interests.
